# Spontaneous sublingual hematoma secondary to warfarin therapy—A rare complication

**DOI:** 10.1002/ccr3.6739

**Published:** 2022-12-13

**Authors:** Aakriti Adhikari, Shriya Sharma, Nirmal Ghimire, Gaurab Mainali, Sumit Kumar Yadav, Ruja Rajkarnikar

**Affiliations:** ^1^ Nepalese Army Institute of Health Sciences Kathmandu Nepal; ^2^ Department of Internal Medicine Nepal Police Hospital Kathmandu Nepal; ^3^ Department of Internal Medicine Shree Birendra Hospital Kathmandu Nepal; ^4^ Kathmandu Medical College and Teaching Hospital Kathmandu Nepal

**Keywords:** anticoagulant, Nepal, sublingual hematoma, warfarin

## Abstract

Warfarin is the most commonly prescribed oral anticoagulant in Nepal. It is commonly used for chronic anticoagulation in patients with atrial fibrillation, venous thromboembolism, and artificial heart valves. The major side effect of warfarin is bleeding. Though extremely rare, a sublingual hematoma can lead to life‐threatening complications as it can cause severe airway obstruction. We present a case of a 55‐year‐old female patient who had sublingual hematoma secondary to the use of Warfarin therapy. In addition to the discontinuation of warfarin, she was managed conservatively without any surgical intervention. Early diagnosis, timely discontinuation of the drug, and application of appropriate medical treatment are of utmost importance for reducing morbidity and mortality due to bleeding and airway compromise.

## Abstract 2

Isolated sublingual hematoma is a rare complication of warfarin treatment. We report a case of a 55‐year‐old woman with complaints of swelling below the tongue and upper neck and difficulty in swallowing and was successfully managed.

## 1 INTRODUCTION

Warfarin is the most commonly prescribed oral anticoagulant, especially in developing countries like Nepal. Despite various novel anticoagulant therapy, it is a major medicine used for chronic anticoagulation in patients with atrial fibrillation (AF), venous thromboembolism (VTE), and artificial heart valves. Warfarin acts as a vitamin K antagonist by binding with the vitamin K 2,3‐epoxide reductase in the hepatic microsome and blocking the action of vitamin K‐dependent factors II, VII, IX, X, protein C, and protein S. Serum warfarin levels are monitored with regular INR with a target of 2–3 in AF and VTE and 2.5–3.5 in patients with mechanical heart valves. There are well‐known hemorrhagic complications of warfarin use. The majority of the complications usually appear in the gastrointestinal, genitourinary, retroperitoneal, and brain parenchyma. There are few reports involving the upper airway. Sublingual hematoma is extremely rare but can lead to potentially life‐threatening complications as severe as airway obstruction. There have been several case reports of such hematoma in the upper airway in anticoagulated patients.^1^, ^2^ We herein present the case of sublingual hematoma secondary to the use of warfarin therapy.

## 2 CASE REPORT

**FIGURE 1:**
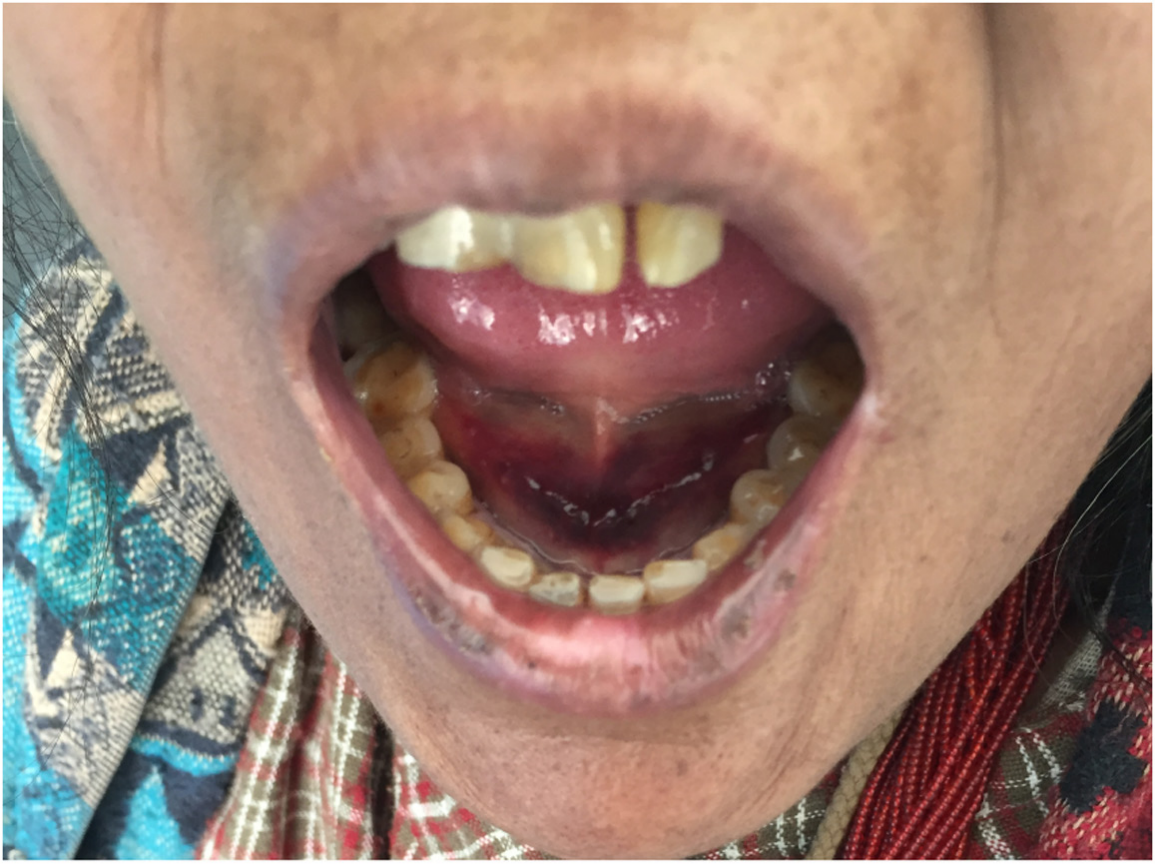
Bleeding and mild swelling under the tongue at the time of admission

**FIGURE 2:**
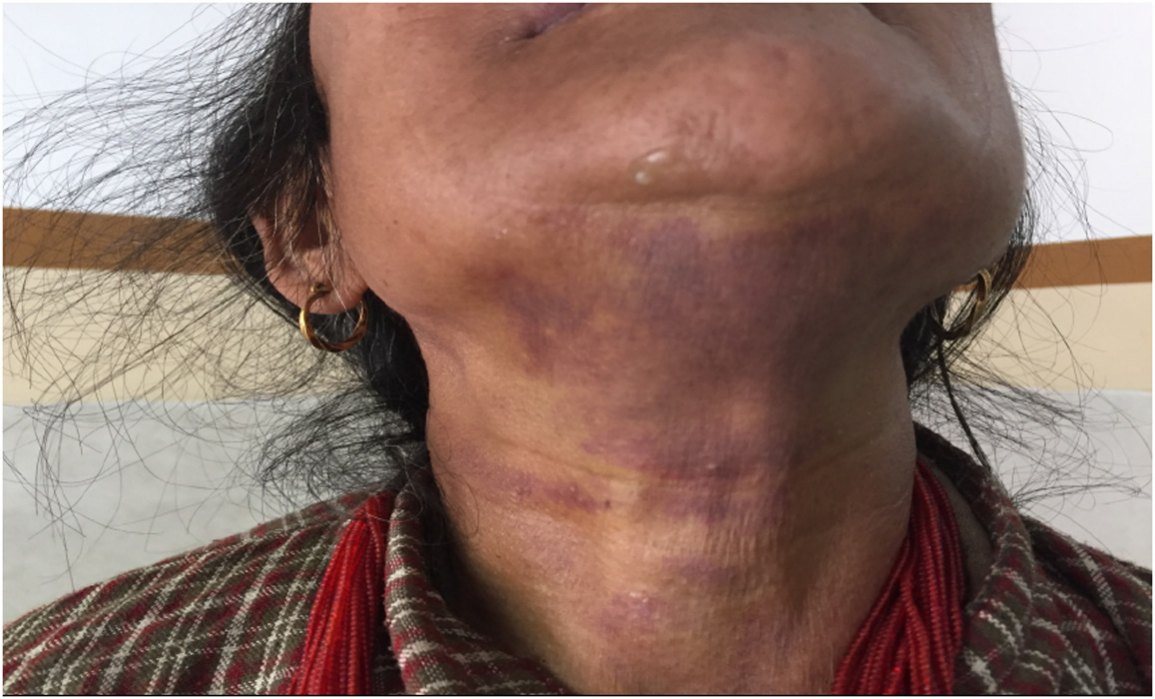
Sublingual hematoma at the time of admission

**FIGURE 3:**
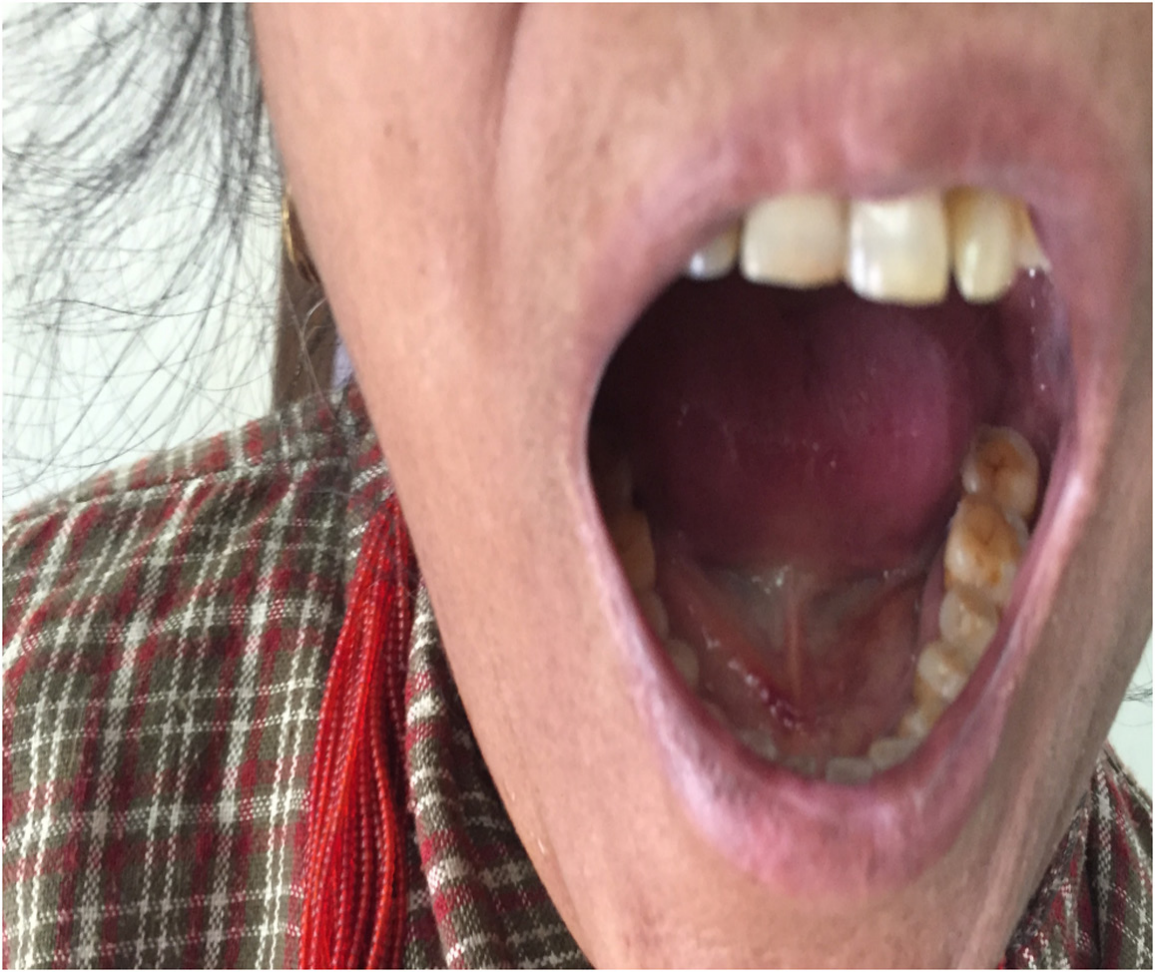
Recovered sublingual hematoma on the day of discharge

A 55‐year‐old female patient visited our emergency ward with complaints of swelling below the tongue and upper neck and difficulty in swallowing for 5 days. She did not give a history of trauma to the area. She had no associated fever, epistaxis, melena, or hematuria. Her medical history included Type 2 Diabetes Mellitus and Mitral valve replacement (mechanical) with Tricuspid valve repair for severe Mitral stenosis and severe Tricuspid Regurgitation secondary to Rheumatic Heart Disease for 2 years. She was on warfarin 10 mg once daily along with other supportive medicines. However, she had stopped taking warfarin and other medicines on her own after developing the abovementioned complaints. The patient's vital signs were normal as suggested as Blood pressure was 130/84 mm of Hg, Heart rate was 90 bpm, Respiratory rate 20/min and, Body temperature 98.8 degree F. General Physical examination was normal except for sublingual hematoma. On arrival at the hospital, her full blood count was normal; however, her INR was recorded as 4.4. The absence of cellulitis, septic markers such as an increased white blood cell count or erythrocyte sedimentation rate, and the grossly elevated INR helped to distinguish this case from Ludwig angina. Diagnosis of sublingual hematoma was made based on clinical presentation, physical examination, and elevated INR. Her warfarin was stopped until her INR returned to normal. Vitamin K and fresh frozen plasma were arranged. She was managed conservatively. She did not require anticoagulant reversal agents. She was closely followed up twice daily in the ward for deterioration of symptoms and meticulous monitoring for the extension of bleeding Figure 1, and Figure 2 was the condition of the patient at the time of admission. However, she improved significantly clinically as shown in Figure 3. and her INR was 1.3 on the 4th day of admission. She was started with warfarin 5 mg. the patient was discharged on the 10th day of admission with a therapeutic range INR of 3.0, achieved with a 10 mg dose of warfarin.

## 3 DISCUSSION

Warfarin is often used as an oral anticoagulant in various conditions like atrial fibrillation or following valvular heart disease.^3^ The mechanism of action of warfarin is to block gamma‐carboxylation of various glutamate residues of vitamin K‐dependent coagulation factors (factor II, VII, IX, X), prothrombin C and S proteins besides with the epoxide reductase activity of vitamin K present in microsomes of the liver.^4^ The majority of complications are bleeding in the structures like gastrointestinal tracts, skin, central nervous system, nose, genitourinary tract, and retroperitoneum. Major bleeding, which includes intracranial hemorrhage and bleeding leading to death or hospitalization, has been reported in 1.2%–8.1% of patients during each year of long‐term warfarin therapy.^3^ The risk of bleeding, internal or external, is related to INR in a log‐linear fashion and is known to be higher with INR levels >4.5.^5^, ^6^ The concurrent usage of platelet‐inhibiting agents such as aspirin and nonsteroidal anti‐inflammatories further increases the risk of bleeding.^5^ Isolated sublingual hematoma is a rare and unexpected complication. There have been many case reports in the medical literature. And these cases differ in their severity.^7^ Sublingual hematoma develops as a result of submucosal bleeding, which can be triggered by minor trauma. They can cause oropharyngeal symptoms and respiratory embarrassment. Recognition of this condition requires a high index of suspicion and a careful history. Sublingual hematoma often develops quickly and rapidly extends posteriorly to involve the surrounding structures like the supraglottic larynx. Patients chiefly complain of discomfort in the throat, drooling, swallowing difficulty, and a change in voice.^8^ In 1976, Lepore mentioned the condition known as a “pseudo‐Ludwig's phenomenon,” which is the result of deranged anticoagulation resulting in spontaneous bleeding in the sublingual and submaxillary spaces.^9^ This leads to elevation of the tongue and floor of the mouth, thus resulting in respiratory distress and complete upper airway obstruction.

In the majority of the previously reported cases, the management of warfarin‐induced sublingual hematoma was largely conservative. The main modalities of treatment were, a reversal of the coagulopathy by the administration of vitamin K and either fresh frozen plasma (FFP), or coagulation‐factor concentrates. Prothrombin complex concentrate is relatively expensive and not easily available in developing countries like Nepal. So, frequently FFP is used. Active vitamin K‐dependent coagulation factors are predominantly in FFP. FFP reverses oral anticoagulant‐induced coagulopathy in most patients. Approximately 15 ml/kg of FFP should be adequate to reverse any coagulopathy. The majority of previously described cases have found a spontaneous resolution of the hematoma once coagulation is normalized.^10^ The first case of successful use of a prothrombin complex concentrate (Beriplex) was described by Lim et al.^11^ Particularly in a sublingual hematoma patient with a history of congestive cardiac failure. It should be taken into consideration that if treatment with warfarin continues, the patient may bleed again and if it is stopped, there is a risk of a thromboembolic phenomenon. Thromboemboli is very low compared to re‐bleeding risk. It is stated that vitamin K or factor replacement is only induced in patients with major bleeding caused by warfarin. Our patient did not need the use of vitamin K and/or FFP as she discontinued warfarin in the very early part of disease onset despite INR of 4.4 at presentation. However, there are many case reports of severe airway obstruction. In some of the reported cases in the medical literature, both invasive (cricothyroidotomy or tracheostomy) and noninvasive (orotracheal intubation) techniques were performed for definitive airway stabilization.^11^, ^12^ In the patients who are considered to be applied endotracheal intubation or surgical airway intervention, the invasive procedure should be performed after anticoagulation is immediately reversed preferably with PCC (50 U/kg). However, the placement of an endotracheal tube due to the obstructive effects of endotracheal intubation of the sublingual hematoma causes the risk of bleeding.^13^ So, timely early discontinuation of anticoagulants and reversal with appropriate agents is crucial for reducing morbidity and mortality of the patients as seen in our patient who even did not require reversal agents due to self‐discontinuation of warfarin by a patient. It is stated that vitamin K or factor replacement is only induced in patients with major bleeding caused by warfarin. Discontinuation of warfarin is recommended in patients with high INR value but without bleeding. Discontinuation of warfarin and follow‐up is recommended in patients with INR value lower than 5 and with bleeding.^14^

## 4 CONCLUSION

Oral anticoagulant drugs like warfarin hold a key role in many cardiac and non‐cardiac conditions. The Patient and his/her relatives should be well informed about the effects of the drug in the sub‐therapeutic, therapeutic, and supra‐therapeutic range. Sublingual hematoma, which is a rare complication should be considered as a differential diagnosis in the patients who receive warfarin treatment and present with complaints of bleeding over and below the tongue, swelling of the neck, dysphagia, and dyspnea. Early diagnosis, timely discontinuation of the drug, and application of appropriate medical treatment are of utmost importance for reducing morbidity and mortality due to bleeding and airway compromise.

## AUTHOR CONTRIBUTIONS

All the authors were involved in the drafting and/or revision of the article and approved the final version to be published.

## FUNDING INFORMATION

This study received no specific grant from any funding agency in the public, commercial, or not‐for‐profit sectors.

## CONFLICT OF INTEREST

The authors declare that they have no competing interest.

## CONSENT

The authors confirm that the patient has provided written informed consent to the submission of this case report, in accordance with the journal's patient consent policy.

## Data Availability

Data are available on request from the authors The data that support the findings of this study are available from the corresponding author upon reasonable request.
